# Prescribers’ experiences regarding deprescribing of polypharmacy at selected district hospitals in Gauteng province, South Africa

**DOI:** 10.4102/hsag.v31i0.3128

**Published:** 2026-01-22

**Authors:** Thabiso R. Phoka, Sisinyana H. Khunou

**Affiliations:** 1 Department of Health Studies, College of Human Sciences, University of South Africa, Pretoria, South Africa

**Keywords:** deprescribing, district hospital, experiences, polypharmacy, prescriber

## Abstract

**Background:**

Polypharmacy is a significant and persistent issue that hinders patient treatment plans and outcomes. It is associated with a wide range of harmful outcomes, including adverse drug interactions, increased morbidity and even mortality rates. The dire effects of polypharmacy have been associated with increased medication errors, hospitalisations, early readmissions and healthcare costs. Despite the extensive documentation and public awareness of these risks, little has been done to address the problem effectively. Deprescribing serves as a valuable strategy to mitigate and prevent the negative consequences associated with polypharmacy.

**Aim:**

The study sought to explore and describe prescriber’s understanding and experiences regarding deprescribing of polypharmacy.

**Settings:**

The study was conducted at district hospitals in Gauteng province.

**Methods:**

A qualitative, descriptive, exploratory and contextual research approach and design was applied in the research. Non-probability purposive sampling techniques were used to select prescribers. Eight steps of data analysis were followed to analyse the data.

**Results:**

Three themes and nine sub-themes emerged from the study: (1) Negative effects of the impact of polypharmacy (the negative impact on the patients, the impact on prescribers and the impact on the organisation), (2) factors that hinder the deprescribing of polypharmacy (prescriber-related factors, patient-related factors, complexity of the prescribing environment and deficiencies in organisations) and (3) measures that can enhance deprescribing of polypharmacy (holistic systems for the deprescribing of polypharmacy and promotion of awareness and education).

**Conclusion:**

This study concluded that prescribers currently believe deprescribing is a complicated process, and few of them actually engage with it. Deprescribing is a safe and effective way to manage inappropriate polypharmacy. This study recommended that improved interdisciplinary and multidisciplinary approaches among health practitioners be introduced to encourage deprescribing, which will avert polypharmacy in the district hospitals.

**Contribution:**

The research findings provide insight, create awareness and encourage safe, rational and judicious prescribing practices among authorised prescribers in district hospitals in Gauteng province.

## Introduction

Polypharmacy first appeared in medical literature more than a century ago and was defined as multiple drug consumption and excessive use (Duerden, Avery & Payne 2019; Sirois et al. 2019). The World Health Organization (WHO 2019) states that the unconventional application of polypharmacy is a global public health challenge. In addition, Turner, Van Vuuren and Leigh-de Rapper (2024) posit that older patients and particularly diabetic patients are at risk for polypharmacy because of co-morbidities. Sub-Saharan Africa also shows similar upward trends. Significantly, a Ugandan study found that polypharmacy was common among human immune-deficiency virus-infected patients who take antiretroviral treatments concurrently with antipsychotic drugs (Eneh et al. 2020). Nwanaji-Enwerem, Boyer and Olufadeji (2021) recommended that health care providers should be trained to educate patients regarding this phenomenon in the African context. According to Shin et al. (2022), polypharmacy has become widespread in most countries, including South Africa. Consistently, the study conducted by Mabulwana and Maaroganye (2025) revealed that the majority of psychiatric patients were on polypharmacy. The study further recommended periodical review of the prescription, deprescribing and health educating patients (Mabulwana & Maaroganye 2025).

Literature has identified the severe effects of polypharmacy. Kamau et al. (2024) revealed that there is a direct correlation between persistent ailments and the prescribing of multiple medicines. Lexow et al. (2021) also accentuate that polypharmacy is linked to hazardous drug effects, which need urgent attention. According to Wylie et al. (2020), apart from ill health, polypharmacy also has dire economic effects, projected at 0.3% of overall health spending. To that effect, Ganga (2022) proposes that countries ought to increase awareness and devise solutions for this problem. In the acknowledgement of complexities associated with polypharmacy, WHO (2019) recommended principles and guidelines to support prescribers. According to Ganga (2022:233), deprescribing plays a crucial role in fostering rational use of medications and enhancing patients’ safety. Bojuwoye, Suleman and Perumal-Pillay (2022) define deprescribing as the procedure of stopping unsuitable drugs from a regimen that is monitored and controlled by a healthcare provider. Despite rising trends in the prevalence and prescribing of polypharmacy in South African public hospitals, the practice of deprescribing remains largely unexplored in the local context (Bojuwoye et al. 2022).

Effective deprescribing requires a collaborative, patient-centred approach that integrates the perspectives of both prescribers and patients. Sawan et al. (2020) emphasise the importance of structured collaboration between healthcare providers and patients to facilitate implementation. Krishnaswami et al. (2019) further underscore the role of trusted relationships and clear communication in supporting patient engagement. However, as Keller, Vordenberg and Steinman (2022) observe, deprescribing in polypharmacy contexts remains complex and under-researched, highlighting the need for more comprehensive evaluation and tailored strategies. Thus, exploring and describing experiences of authorised prescribers from two selected district hospitals has assisted in better understanding the experiences of prescribers about polypharmacy and the use of deprescribing as a tool to avert it. The purpose of the study was to explore and describe the experiences of the prescribers in deprescribing polypharmacy.

## Research methods and design

The research applied a qualitative exploratory contextual design, which focused on real human experiences, meaning and understanding from the point of view of authorised prescribers from selected district hospitals in Gauteng province. The findings obtained during data collection unpacked a dense description of several experiences regarding deprescribing of polypharmacy.

### Population and sampling

The study included participants who were authorised to prescribe, with 2 years of working experience in the two selected hospitals. The researcher recruited the participants by conducting several meetings and presented the study proposal. Prescribers included community service doctors, medical officers, specialists and consultants. These prescribers are an ideal target in the study as they play a key role in the prescribing and initiation of polypharmacy and, as such, bear significant responsibility in deprescribing. Purposive non-random sampling was used to gain an in-depth understanding from 13 authorised prescribers. Individual face-to-face, in-depth, semi-structured interviews were used to collect data from 13 participants.

### Data collection

The researcher was responsible for data collection by conducting individual semi-structured interviews. The interviews were conducted in English in a private, designated, noise-free room from August to October 2023 and lasted for 30 to 103 min. The interview guide was used to obtain data from the participants. Three focal questions were used during the interview: ‘What are your views regarding deprescribing as a tool to avert polypharmacy?’; ‘According to your observation, what are the factors that contribute to polypharmacy?’ and ‘What do you think can be done to enhance deprescribing of polypharmacy?’ Extra probing questions were asked to gather a deeper understanding of polypharmacy. The interviews were captured by the researcher with the use of an audio recorder with the permission of the participants. Field notes were documented by the researcher in order to capture the nonverbal cues displayed by the participants. The interviews continued until data saturation was reached at participant number 13, when additional data no longer yielded new insights. Data collected by the researcher was transcribed verbatim, then submitted to the independent co-coder to confirm and enhance the trustworthiness of the results.

### Data analysis

Data were analysed manually, using Tesch’s eight steps of qualitative data analysis as outlined in Cresswell and Cresswell (2018) and Makua and Khunou (2022). The researcher commenced by reading the entire transcripts in order to get an understanding of the underlying meaning. Individual transcripts were read to gain insight into the developing subtopics, which were then recorded in the document margin. Broad topics were outlined and summarised with codes that were duly outlined in relevant areas. In step number five, descriptive words were aligned with the identified topics and categorised into groups. The categories were then summarised and named appropriately to avoid repetition. The last step included documentation to get the comprehensive meaning and general sense of the data.

### Trustworthiness

Validation of data was carried out by a qualified co-coder who was outsourced to independently code the qualitative data using the provided protocols. Both the researcher and the co-coder arrived at an agreement for the good and purpose of achieving conformability and thereby enhancing trustworthiness. Probing, clarifying and paraphrasing techniques were employed to allow free expression until data saturation was reached. Field notes were documented, and audio recordings were used to capture the data, which was transcribed verbatim. Credibility was achieved by meaningfully engaging with the participants. The researcher established a positive rapport and encouraged free engagement. Verification permitted participants to rectify errors and volunteer additional information. Following the transcription process, the researcher presented the transcribed notes to the participants. To achieve dependability, care was taken to ensure that the study methods were verifiable, logical and explicitly documented in a succinct manner by giving a detailed description of the study methods.

### Ethical considerations

The study obtained ethical clearance from UNISA’s Higher Degree Ethics Committee (Reference number: REC-248016-052). Permissions were also obtained from the Department of Health Johannesburg Health District and Ekurhuleni Health District Research Committees (Reference number: 202308-004). The researcher adhered to confidentiality and respect for the participants’ rights. The researcher signed a confidentiality form, which bound them to comply with and adhere to privacy and confidentiality clauses pertaining to qualitative research. The researcher adhered to principles of respect, beneficence and justice throughout the study (Polit & Beck 2017).

## Results

Thirteen participants (see Table 1) met the inclusion criteria and were subsequently interviewed. The results are reported in the next section.

**TABLE 1:** Demographic characteristics of the participants.

Participant	Age in years	Gender	Race
P1	34	Female	African person
P2	48	Male	Indian person
P3	45	Male	African person
P4	33	Male	African person
P5	34	Female	African person
P6	35	Female	African person
P7	34	Female	African person
P8	35	Male	African person
P9	49	Male	African person
P10	30	Female	Indian person
P11	34	Female	White person
P12	40	Female	African person
P13	50	Male	African person

**FIGURE 1:**
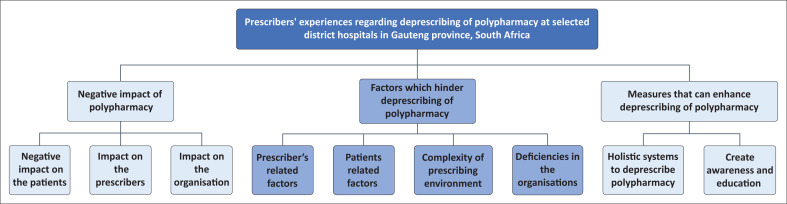
Themes and sub-themes revealed by the study.

The participants were categorised into the following three age groups: 25 years – 35 years, 36 years – years 49 years and 50 years and above. The number of participants between the ages 25 years – 35 years was eight (*n* = 8; 62%), between 36 years and 49 years was four (*n* = 4; 31%) and participants above the age of 50 years and above was one (*n* = 1; 7%). The participants’ gender was constituted by seven (*n* = 7) females and six (*n* = 6) males. The level of training was constituted by 10 medical officers (*n* = 10), two specialists (*n* = 2) and one consultant (*n* = 1). The participant’s years of experience ranged from 2 years to 20 years. Seven prescribers had 3 years – 5 years of experience, two prescribers had 5 years – 10 years of experience and four prescribers had 10 years – 20 years of experience. This section presents research findings on prescribers’ understanding of polypharmacy and their experiences regarding deprescribing of polypharmacy. This study yielded three themes in the data analysis. The themes and categories of the study are summarised in Figure 1. Each theme has categories described with the following direct quotes from the study participants.

### Theme 1: Negative impact of polypharmacy

The study revealed the negative impacts of polypharmacy as addressed under the following emergent sub-themes, namely: (1) the negative impact on the patients, (2) the impact on prescribers and (3) the impact on the organisation.

#### Sub-themes 1.1: The negative impact on the patients

The impacts on the patient are numerous and perpetuated by prescribing polypharmacy. These are increased adverse effects and events, poor adherence, medication misuse, disuse, abuse, increased medication errors, increased readmission rates soon after discharge and generally poor prognosis. The following extracts attest to this:

‘It’s almost a lot of the patients are like on 30-day course of Benzodiazepines, and you know it’s meant to be for like five days only and stop after a week but these patients are on it for years so to stop it it’s a problem, they will pick me up or strangle me, so they like addicted to it [*Worried looking*].’ (P10)

‘Yes, so what I’ve seen now, post covid, the pandemic had a huge impact on mental health, uhm … [*Pondering*] … with people losing jobs and not being able to cope mentally and it does, it appeals to you, for the doctor or a healthcare worker, you see someone who is struggling to sleep. Sleep is something that we should be doing, you are trying to do well, you say as doctor maybe let me start you on this tryptanol, to help you sleep. Then come next month … “Doctor that thing you gave me really works. I need some of that again …” so they get hooked … [*chuckles*].’ (P3)

#### Sub-themes 1.2: Impact on the prescribers

An overwhelming majority of participants cited the enormous workload they face on a daily basis. Many prescribers identified stress as a contributing factor. This challenge had a cascading effect because an increase in disproportional doctor–patient ratios leads to escalations in workload. This resulted in a decrease in the amount of time available for deprescribing, thereby increasing the stress levels of the prescribers. The following quotes support this:

‘So I can say the workload also contributes to polypharmacy. We spend 5–10 minutes and off the patient goes. So, it starts with us. It needs time and more work force.’ (P9)

‘Uuhm … it’s easier for a doctor to just write a long script just to get a patient to leave than to sit and discuss with a patient, what they need and whatever cause there too many patients you see.’ (P7)

#### Sub-themes 1.3: Impact on the organisation

The Department of Health has provided comprehensive guidelines, legislative frameworks and SOPs that outline the functions and governance of public health facilities. Organisational effectiveness is imperative in preventing polypharmacy. Participants expressed that the health facilities incur huge amount of financial burden as a result of polypharmacy:

‘To be honest cost don’t cross my mind, I prescribe on clinical guidelines I don’t think about how much is it costing. I think I would consider cost if patients had access to a lot more better stuff so maybe I’ll start to think if you giving this then maybe don’t give that but as I said here we have very minimal things for certain conditions, can cost be really a factor when you have given us such little access to medication.’ (P6)

### Theme 2: Factors that hinder deprescribing of polypharmacy

The factors that discourage the deprescribing of polypharmacy were the second theme that emerged. This theme gave rise to four sub-themes: (1) prescriber-related factors, (2) patient-related factors, (3) complexity of the prescribing environment and (4) deficiencies in organisations.

#### Sub-themes 2.1: Prescriber-related factors

The prescribers voiced concerns about the knowledge, attitude and power relations that exist in the deprescribing of polypharmacy according to their lived experiences. This was highlighted by the following extracts:

‘Sometimes we lack knowledge when it comes to drugs and how they act, so like if I prescribe a particular drug I feel that it won’t be sufficient for that particular condition, so I add another drug. Sometimes you adopt those tendencies from seniors or those who have been in the department for long. You see what they do and you adopt. But sometimes it could be the patient’s condition itself, like it’s difficult to treat with 1 agent or sometimes the patient has multiple comorbidities. Therefore, you have to treat all of them, not just one.’ (P5)

#### Sub-themes 2.2: Patient-related factors

The participants expressed their views on factors in their daily functioning, which have impacts that mitigate or discourage deprescribing of polypharmacy from the patient’s perspective. The following extracts support this:

‘The patients themselves are resistant to change their medications. They just work on previous history that it was there. They not looking at why the medication is not there anymore they want it back on.’ (P1)

#### Sub-themes 2.3: Complexity of prescribing environment

The prescribers highlighted that because of the varied nature of prescribing environments in the health care sector, it was somewhat complicated to deprescribe. Issues of referrals and levels of healthcare came to the fore. The following excerpt reveals this:

‘A lot of these patients are from private and they are on high doses and when you tell them you need to reduce their doses, they will fight you, they will stand up from the chair and throw tantrums. And it’s not just one patient, they will tell you they won’t sleep at night, they’ll tell you they won’t be able to work, they can’t function etc. So, like I said yes, the patients’ attitudes need to change, more education is needed, these patients if you resist giving them something they will even stand up and shout I tell you, they can’t live without it.’ (P10)

#### Sub-themes 2.4: Deficiencies in the organisations

The prescribers pointed out that there are pre-existing organisational challenges and insufficiencies that promote polypharmacy prescribing and discourage deprescribing. It emerged that working conditions and tools that may enable prescribers to work efficiently and effectively are deficient in the health facilities. The following quotes attest to this:

‘There are fewer doctors and too many patients and we force to push them out as quick as possible. That is why we miss a lot of things an opportunity to deprescribe for example precisely because of the minimal time we have.’ (P11)

‘Giving access to better medications as much as it’s available in private. And I think that will help, it might seem costly Initially, but it will help with reducing cost; long term because I don’t have to give a patient five medications whereas I can give them one and then you end up saving. So, give me good pain medication. Give the top of the range hypertensive, diabetic. Give as much as you can of the best stuff we can reduce polypharmacy. I think that’s big thing for me, we don’t have access here in the district hospital.’ (P6)

### Theme 3: Measures that can enhance deprescribing of polypharmacy

The prescribers recommended remedial and preventative measures to avert polypharmacy. This theme led to the development of two distinct categories: (1) holistic systems for the deprescribing of polypharmacy and (2) promotion of awareness and education.

#### Sub-themes 3.1: Holistic systems to deprescribe polypharmacy

The success of deprescribing polypharmacy is dependent on the extent to which all stakeholders work together advantageously and synergistically to achieve a common goal. As revealed in the engagements, deprescribing requires a multifaceted approach involving patients, doctors, pharmacists, administrators, management and caregivers. The following extracts support this:

‘Appropriate referrals and adhering to guidelines. There’s algorithms and tables we look at when prescribing for common diseases. For example, for hypertensives, they are on four agents before they advise you to refer and you’ve got to maximise the doses as well. So that’s at least six months of prescription before we can initiate referrals.’ (P6)

#### Sub-themes 3.2: Create awareness and education

The prescribers felt that creating awareness and conscientisation towards the concept of deprescribing was vitally important to curb the prevalence of polypharmacy. Two categories emerged from this sub-theme namely: Health education to patients and development of the prescribers. The following quotes speaks to these:

‘So the number one thing is patient education. Patient education, prevention of those ailments and moving away from this treatment mentality that you know in the state, if you check research, people are being weaned off medication just with good exercise, good sleep, good eating habits, people are being removed from diabetic treatment, so you eat well, sleep well, you exercise, you get away from this polypharmacy but we are in a setting, our circumstances don’t allow us, I can’t tell you don’t eat mealie meal every day because it’s our staple and what we can afford.’ (P3)

## Discussion

The study aimed to explore and describe experiences of authorised prescribers regarding deprescribing of polypharmacy at selected district hospitals in Gauteng. Three themes with supporting sub-themes emerged from the participants’ understanding of the concept of polypharmacy and experiences pertaining to deprescribing of polypharmacy. The findings obtained during the course of data collection unpacked dense description of several experiences regarding deprescribing of polypharmacy. From the study’s findings, it has been revealed that the majority of the participants did have a fair understanding of the concept of polypharmacy and were aware of the negative impacts thereof. In addition, some were aware of deprescribing; however, not a lot were engaged with the process on a regular basis in the selected district hospitals in Gauteng province. Theme 1 deliberated on the negative impacts of polypharmacy. Theme 2 unpacked factors that hinder deprescribing of polypharmacy, while theme 3 highlighted measures that can enhance deprescribing of polypharmacy.

### Negative impact of polypharmacy

The prescribers described that polypharmacy has a negative impact on the patients, prescribers and the organisation. It is clear that the aftermath of polypharmacy on patients includes drug abuse and adverse drug effects. In most cases, prescribers found an addiction to antipsychotic drugs among other prescribed medications. The study’s findings were in agreement with a study by Singier et al. (2021) who articulate that misuse of drug is unwarranted if it does not comply with regulatory and medical guidelines. Furthermore, drug misuse and abuse are linked to increased risk of adverse reactions, addiction and fatal overdose. In addition, Karakaş et al. (2019) also confirmed that medicine exploitation, addiction and misuse are common in day-to-day medical practice. The participants admitted to having difficulties distinguishing between medical ailments and symptoms because of the side effects of medications. According to WHO (2019), an ADR is a harmful and unexpected response to a medicine, occurring at doses typically used for diagnosis, disease therapy, prophylaxis or modification of physiological functions. In this study, it was outlined that an increase in ADRs resulted in additional demand for healthcare utilisation. To corroborate, Osanlou et al. (2022) explore adverse drug reactions, multimorbidity and polypharmacy, highlighted that there were 218 identified patient admissions with an ADR giving a prevalence of 18.4%. To support, Pereira, Veríssimo and Ribeiro (2024) assert that there exists a direct correlation between polypharmacy and increased adverse drug reactions.

Because of polypharmacy, prescribers bored the brunt of work pressures as a result of polypharmacy. In this aspect, the prescribers had to spend most time dealing with the effects of polypharmacy, be it the ADR or misuse and addiction of drugs. In the same breath, the health organisation had to spend a huge amount of money in dealing with polypharmacy. In this regard, prescribers attributed the need for more healthcare resources in order to manage polypharmacy. As a remedial action, Akande-Sholabi, Ajilore and Ilori (2023) submit that adequate resources should be availed in order to avert ADRs through judicious deprescribing. This may demonstrate that prescribers have proactive attitudes and the intention to alleviate negative effects of drugs.

### Factors that hinder deprescribing of polypharmacy

This section addresses objective two of the study, which deals with experiences of prescribers regarding deprescribing of polypharmacy. Prescribers cited a negative attitude towards deprescribing because practitioners do not want to upset patients by removing some of the medications from their long list. This was in line with Giménez et al. (2020) who explored prescriber’s attitudes and beliefs about polypharmacy and found that there are positive attitudes, and some prescribers do in fact make deliberate efforts to reduce polypharmacy. In addition, this can be attributed to the lack of confidence and knowledge about medications and guidelines. Thus, the experience of prescribers’ reveals apathy, demotivation, discouragement and fear to deprescribe, which are all negative attributes. The lack of pharmacological knowledge was also alluded to in the study. The participants indicated that their prescribing tendencies are informed by what is done in the system by their seniors. It is interesting to note that people who are supposed to be knowledgeable about prescribing medicines are not proficient or well acquainted with the pharmacology of medicines. To corroborate, Mahomedradja et al. (2023) postulate that prescribing errors occur in 7% of total medication orders and up to 50% of hospital admissions.

Prescribers also pointed out several organisational barriers that hindered the deprescribing of polypharmacy. The absence of deprescribing guidelines and protocols significantly hinder the process of deprescribing. This underpinned by a study by Bashkin et al. (2022) highlighting that doctors explained that prevalent lack of expertise and proficiency points to an urgent need for upscaling of skills to deal with difficulties such as deprescribing polypharmacy. Furthermore, Davila et al. (2022) advise that refining admittance to easy technologies as tools for deprescribing will aid the process.

Most participants gave accounts of how busy the hospitals and clinics are, and that most prescribers are overworked, making it difficult to include deprescribing into a consultation. Some participants expressed concerns about the absence of beneficial treatment alternatives that could prevent polypharmacy. To corroborate this, prescribers expressed that the diversity of prescribers, each with their own philosophy regarding medication prescribing, presents a significant challenge on deciding the prescriber supports medication discontinuation or not. The prescribers stressed that patients resist the suggestion to take less medication. In their minds, more medication means better health; when they have become accustomed to taking a lot, they are not keen to change.

### Measures that can enhance deprescribing of polypharmacy

Participants expressed the need for a holistic system to deprescribe polypharmacy through inculcation of guidelines, SOPs, creating awareness and education at both prescriber and patient level. To fortify, Anderson et al. (2024) confirm that most systemic impediments can be attributed to a lack of information on a patient’s full medical records and previous diagnoses, prognosis indications and medicinal regimen. It is important for prescribers to know when and how to up-refer and down-refer a patient. Participants expressed the importance of raising awareness and educating prescribers about the concept of deprescribing. A resounding agreement by majority of participants emphasised the crucial role of patient education and counselling to encourage patients to consider deprescribing polypharmacy as a viable option.

Participants expressed that although their medical training might have been reasonably good, it did not put emphasis on pharmacology and pharmaceutics as important subjects but rather focussed on diagnosing and examining practice and technique. To corroborate, Trani et al. (2024) suggest that enhancing educational programme should include the following: elicit patients’ specific needs prior to intervention and delivery, facilitating the provision of concise summaries and key messages, implementing regular follow-ups with participant’s post-intervention and incorporating family members into educational sessions.

In that regard, it was addressed that despite the existence of several evidence-based deprescribing tools and guidelines, the limited awareness highlights the need for further training and sensitisation to enhance knowledge and equip doctors with the much-needed skills to deal with the existing demands of modern-day multimorbidities. Findings by Akande-Sholabi et al. (2023) affirm that there is a dearth of knowledge on deprescribing tools and guidelines, and this needs to be rectified. In addition, medical education offered by university medical schools should make a concerted effort to inculcate deprescribing of polypharmacy as part of a learning objective. Furthermore, Omer et al. (2021) advises that it is crucial for medical students to improve their theoretical knowledge alongside the development of necessary skills and attitudes for safe prescribing within an interactive clinical context.

### Recommendations

Enhance drug information services by establishing a mechanism for medication error identification and reporting. Interventions targeting professional development, better collaboration structures with laboratories and clearer and more user-friendly guidelines could potentially support rational antibiotic prescribing behaviour. In addition, better networking and social support among physicians could support lower prescription rates. Improved knowledge on deprescribing and inclusion as a subject matter for continuous professional development. Research must be conducted to develop the deprescribing instruments specifically designed for public hospitals. More researches are required to establish the level of adherence to prescribing and deprescribing guidelines. It would also assist in investigating prescribers’ knowledge and confidence in deprescribing guidelines. An exploration of prescriber-related factors influencing deprescribing within a multidisciplinary team.

## Conclusion

The main aim of this study was to explore and describe the experiences of authorised prescribers at two selected district hospitals. The goal was to help prescribers better understand their experiences with polypharmacy and how to use deprescribing to avert it. Polypharmacy reduction will help to alleviate the medication burden, which frequently results in undesirable side effects, increased hospitalisation, non-compliance, high medical costs, drug complications and in some cases death. Deprescribing is the most definitive and effective way to circumvent the ills of polypharmacy. The greater the familiarity and adherence of prescribers to the guidelines, the better the healthcare system’s outcomes. The dichotomy of polypharmacy prescribing will be ameliorated by applying deprescribing as a tool to avert incoherencies, inconsistencies, and anomalies in practice.

## References

[CIT0001] Akande-Sholabi, W., Ajilore, C.O. & Ilori, T., 2023, ‘Evaluation of physicians’ knowledge of deprescribing, deprescribing tools and assessment of factors affecting deprescribing process’, *BioMed Central Primary Care* 24(1), 31. 10.1186/s12875-023-01990-1 PMC9875427 36698057

[CIT0002] Anderson, T.S., Wang, B.X., Lindenberg, J.H., Herzig, S.J., Berens, D.M. & Schonberg, M.A., 2024, ‘Older adult and primary care practitioner perspectives on using, prescribing, and deprescribing opioids for chronic pain’, *JAMA Network Open* 7(3), e241342. 10.1001/jamanetworkopen.2024.1342 38446478 PMC10918495

[CIT0003] Bashkin, O., Otok, R., Kapra, O., Czabanowska, K., Barach, P., Baron-Epel, O. et al., 2022, ‘Identifying the gaps between public health training and practice: A workforce competencies comparative analysis’, *International Journal of Public Health* 67, 1605303. 10.3389/ijph.2022.1605303 36618436 PMC9812945

[CIT0004] Bojuwoye, A.O., Suleman, F. & Perumal-Pillay, V.A., 2022, ‘Polypharmacy and the occurrence of potential drug–drug interactions among geriatric patients at the outpatient pharmacy department of a regional hospital in Durban, South Africa’, *Journal of Pharmaceutical Policy and Practice* 15(1), 1. 10.1186/s40545-021-00401-z 34983680 PMC8729144

[CIT0005] Creswell, J.W. & Creswell, J.D., 2018, *Research* design: *Qualitative, quantitative, and mixed methods approach*, 5th edn., Sage Publications, Thousand Oaks, CA.

[CIT0006] Davila, H., Rosen, A.K., Stolzmann, K., Zhang, L. & Linsky, A.M., 2022, ‘Factors influencing providers’ willingness to deprescribe medications’, *Journal of the American College of Clinical Pharmacy* 5, 15–25. 10.1002/jac5.1537

[CIT0007] Duerden, M., Avery, T. & Payne, R.A., 2019, *Polypharmacy and medicines optimization: Making it safe and sound*, The King’s Fund, London.

[CIT0008] Eneh, P.C., Huppler Hullsiek, K., Kiiza, D., Rhein, J., Meya, D.B., Boulware, D.R. et al., 2020, ‘Prevalence and nature of potential drug-drug interactions among hospitalized HIV patients presenting with suspected meningitis in Uganda’, *BioMed Central Infectious Diseases* 20, 572. 10.1186/s12879-020-05296-w 32758158 PMC7405463

[CIT0009] Ganga, N.P., 2022, ‘Polypharmacy: An overview’, *Journal of Pharmacy Care* 10(4), 229–233.

[CIT0010] Giménez, S., Anciano, M., Cortés, J., Ramos, M., Pizarraya, M. & Verdug, P., 2020, ‘Beliefs and attitudes about deprescription in older HIV-infected patients: ICARD Project’, *Sociedad Española de Quimioterapia* 34(1), 18–27. 10.37201/req/084.2020 PMC7876903 33191724

[CIT0011] Karakaş Uğurlu, G., Uğurlu, M., Sereyim, S. & Çayköylü, A., 2019, ‘A brief review of misuse, abuse, addiction, and pseudo-addiction concepts through a case’, *Psychiatria Danubina* 31(3), 360–361. 10.24869/psyd.2019.360 31596831

[CIT0012] Keller, M.S., Vordenberg, S.E. & Steinman, M.A., 2022, ‘Health services research’, *Journal of General Internal Medicine* 37(12), 3176–3177. 10.1007/s11606-022-07537-x 35411528 PMC9485366

[CIT0013] Krishnaswami, A., Steinman, M., Goyal, P., Zullo, A.R., Anderson, T.S., Birtcher, K.K. et al., 2019, ‘Deprescribing in older adults with cardiovascular disease’, *Journal of the American College of Cardiology* 73(20), 2584–2595. 10.1016/j.jacc.2019.03.467 31118153 PMC6724706

[CIT0014] Lexow, M., Wernecke, K., Schmid, G.L., Sultzer, R., Bertsche, T. & Schiek, S., 2021, ‘Considering additive effects of polypharmacy: Analysis of adverse events in geriatric patients in long-term care facilities’, *Wiener Klinische Wochenschrift* 133, 15–16. 10.1007/s00508-020-01750-6 PMC8373749 33090261

[CIT0015] Mabulwana, L.-A. & Maaroganye, K., 2025, ‘Complex Psychotropic Polypharmacy in Sowetobased community psychiatry clinics’, *South African Journal of Psychiatry* 31, a2424. 10.4102/sajpsychiatry.v31i0.2424 PMC12339778 40799259

[CIT0016] Mahomedradja, R.F., Schinkel, M., Sigaloff, K.C.E., Reumerman, M.O., Otten, R.H.J., Tichelaar, J. et al, 2023, ‘Factors influencing in-hospital prescribing errors: A systematic review’, *British Journal of Clinical Pharmacology* 89(6), 1724–1735. 10.1111/bcp.15694 36805648

[CIT0017] Makua, S.R. & Khunou, S.H., 2022, ‘Nurse managers’ views regarding patients’ long waiting time at community health centers in Gauteng Province, South Africa’, *Belitung Nursing Journal* 8(4), 325–332. 10.33546/bnj.2096 37546493 PMC10401372

[CIT0018] Nwanaji-Enwerem, J.C., Boyer, E.W. & Olufadeji, A., 2021, ‘Polypharmacy exposure, aging populations, and COVID-19: Considerations for healthcare providers and public health practitioners in Africa’, *International Journal of Environmental Research Public Health* 18, 10263. 10.3390/ijerph181910263 34639561 PMC8507838

[CIT0019] Omer, U., Danopoulos, E., Veysey, M., Crampton, P. & Finn, G., 2021, ‘A rapid review of prescribing education interventions’, *Medical Science Education* 31(1), 273–289. 10.1007/s40670-020-01131-8 PMC8368780 34457882

[CIT0020] Osanlou, R., Walker, L., Hughes, D.A., Burnside, G. & Pirmohamed, M., 2022, ‘Adverse drug reactions, multimorbidity and polypharmacy: A prospective analysis of 1 month of medical admissions’, *BioMedical Journal Open* 12(7), e055551. 10.1136/bmjopen-2021-055551 PMC9255409 35788071

[CIT0021] Pereira, A., Veríssimo, M. & Ribeiro, O., 2024, ‘Influence of chronic medical conditions on older patients’ willingness to deprescribe medications: A cross-sectional study’, *BioMed Central Geriatrics* 24(1), 315. 10.1186/s12877-024-04891-9 38575904 PMC10993447

[CIT0022] Polit, D.F. & Beck, C.T., 2017, *Essentials of nursing research: Appraising evidence for nursing practice*, 10th edn., JB Lippincott Williams & Wilkins, Philadelphia, PA.

[CIT0023] Rozsnyai, Z., Jungo, K.T., Reeve, E., Poortvliet, R.K.E., Rodondi, N., Gussekloo, J. et al., 2020, ‘What do older adults with multi-morbidity and polypharmacy think about deprescribing? The LESS study – A primary care-based survey’, *BioMed Central Geriatrics* 20(1), 1–11. 10.1186/s12877-020-01843-x PMC7602330 33129274

[CIT0024] Sawan, M., Reeve, E., Turner, J., Todd, A., Steinman, M.A., Petrovic, M. et al., 2020, ‘A systems approach to identifying the challenges of implementing deprescribing in older adults across different health care settings and countries: A narrative review’, *Expert Review of Clinical Pharmacology* 13(3), 233–245. 10.1080/17512433.2020.1730812 32056451 PMC7309419

[CIT0025] Shin, W.Y., Go, T.H., Kang, D.R., Lee, Y., Lee, W., Lee, K.L. et al., 2022, ‘Patterns of patients with polypharmacy in the adult population from Korea’, *Scientific Reports* 12, 18073. 10.1038/s41598-022-23032-z 36302935 PMC9613698

[CIT0026] Singier, A., Noize, P., Berdaï, D., Daveluy, A., Arnaud, M., Molimard, M. et al., 2021, ‘Medicine misuse: A systematic review and proposed hierarchical terminology’, *British Journal of Clinical Pharmacology* 87(4), 1695–1704. 10.1111/bcp.14604 33295072

[CIT0027] Sirois, C., Domingues, N.S., Laroche, M.L., Zongo, A., Lunghi, C., Guénette, L. et al., 2019, ‘Polypharmacy definitions for multimorbid older adults need stronger foundations to guide research, clinical practice, and public health’, *Pharmacy (Basel)* 7(3), 126. 10.3390/pharmacy7030126 31470621 PMC6789889

[CIT0028] Trani, M.R., Bilocura, I., Bersabal, S., Panilagao, R.K., Toledo, B.R., Garrido, E. et al., 2024, ‘Effects of a comprehensive structured patient education intervention on disease-related knowledge and behaviour change among people living with type 2 diabetes in the Philippines’, *Frontiers in Rehabilitation Sciences* 5, 1374850. 10.3389/fresc.2024.1374850 38481977 PMC10933123

[CIT0029] Turner, M.J., Van Vuuren, S. & Leigh-de Rapper, S., 2024, ‘Analysing patient factors and treatment impact on diabetic foot ulcers in South Africa’, *South African Journal of Science* 120(3/4), Art. #16301. 10.17159/sajs.2024/16301

[CIT0030] World Health Organization, 2019, *Medication safety in polypharmacy* (WHO/UHC/SDS/2019.11), WHO, Geneva. Licence: CC BY-NC-SA 3.0 IGO.

[CIT0031] Wylie, C.E., Daniels, B., Brett, J., Pearson, S.A. & Buckley, N.A., 2020, ‘A national study on prescribed medicine use in Australia on a typical day’, *Pharmacoepidemiology and Drug Safety* 29(10), 1046–1053. 10.1002/pds.5093 32779806

